# THE EVOLUTION OF A PUBLIC HEALTH APPROACH TO ADRD AND THE ROLE OF RISK REDUCTION

**DOI:** 10.1093/geroni/igac059.510

**Published:** 2022-12-20

**Authors:** Lisa McGuire, Eva Jackson

**Affiliations:** CDC, Atlanta, Georgia, United States; Alzheimer's Association, Chicago, Illinois, United States

## Abstract

CDC, through the Healthy Brain Initiative (HBI) and Building Our Largest Dementia (BOLD) Infrastructure Act, is working to advance cognitive and brain health as integral components of public health practice, to keep older Americans healthy and independent as long as possible. HBI promotes brain health as part of public health practice and BOLD strives to build a uniform public health infrastructure. Both HBI and BOLD focus not only on people with cognitive decline or dementia but also their health care providers and caregivers. Recently, the 2021 Alzheimer’s Disease National Plan added Goal 6, Accelerate Action to Promote Healthy Aging and Reduce Risk Factors for Alzheimer’s Disease and Related Dementias. This presentation will describe the evolution of public health’s role with respect to brain health and caregiving and how the national priority on risk reduction and healthy aging can be beneficial to the health and well-being of older adults.

